# Tumor Treating Fields (TTFields) Induce Cell Junction Alterations in a Human 3D In Vitro Model of the Blood-Brain Barrier

**DOI:** 10.3390/pharmaceutics15010185

**Published:** 2023-01-04

**Authors:** Ellaine Salvador, Theresa Köppl, Julia Hörmann, Sebastian Schönhärl, Polina Bugaeva, Almuth F. Kessler, Malgorzata Burek, Ralf-Ingo Ernestus, Mario Löhr, Carsten Hagemann

**Affiliations:** 1 Section Experimental Neurosurgery, Department of Neurosurgery, University Hospital Würzburg, 97080 Würzburg, Germany; 2 Department of Anesthesiology, Intensive Care, Emergency and Pain Medicine, University Hospital Würzburg, 97080 Würzburg, Germany

**Keywords:** blood-brain barrier, Tumor-Treating Fields (TTFields), CNS disorders, human brain microvascular endothelial cells (HBMVEC), human cells, 3D in vitro model

## Abstract

In a recent study, we showed in an in vitro murine cerebellar microvascular endothelial cell (cerebEND) model as well as in vivo in rats that Tumor-Treating Fields (TTFields) reversibly open the blood–brain barrier (BBB). This process is facilitated by delocalizing tight junction proteins such as claudin-5 from the membrane to the cytoplasm. In investigating the possibility that the same effects could be observed in human-derived cells, a 3D co-culture model of the BBB was established consisting of primary microvascular brain endothelial cells (HBMVEC) and immortalized pericytes, both of human origin. The TTFields at a frequency of 100 kHz administered for 72 h increased the permeability of our human-derived BBB model. The integrity of the BBB had already recovered 48 h post-TTFields, which is earlier than that observed in cerebEND. The data presented herein validate the previously observed effects of TTFields in murine models. Moreover, due to the fact that human cell-based in vitro models more closely resemble patient-derived entities, our findings are highly relevant for pre-clinical studies.

## 1. Introduction

In the treatment of brain tumors, a persistent hurdle that even the most potent chemotherapeutics have to encounter is the restrictiveness of the blood-brain barrier (BBB). The ability of drugs to reach the tumor site mostly depends on their being able to cross the BBB, which is mostly made up of endothelial cells sealed by tight junctions (TJs) and supported by pericytes, astrocytes, and neurons [1,2,3,4]. However, the BBB restricts the passage of drugs; as a result, the quest for novel modalities to increase the permeation of these drugs is necessary for their success in treating CNS tumors.

The restrictiveness of the BBB is attributed to TJs made up of multiprotein complexes such as the family of claudins, occludin, and tricellulin [5,6]. Transmembrane TJs are linked to the cytoskeleton through scaffolding proteins, such as Zonula occludens (ZO)-1 [7]. Further, TJs are regulators of water, ion, and molecular transport through the paracellular pathway and help maintain vascular homeostasis [8]. In addition to TJs, the integrity of the barrier is regulated by the platelet endothelial cell adhesion molecule (PECAM)-1 [9].

The existing in vitro models of the BBB range from simple monolayers of endothelial cells to more complex transwell and microfluidic systems [10,11,12]. The complexity of the model lies in its physical construction as well as the number and types of cells used for it. There are models that use up to four cell types, namely endothelial cells, pericytes, astrocytes, and neurons [12,13,14,15,16]. However, several models employ human-induced pluripotent stem cells (iPSCs)-derived human brain microvascular endothelial cells, one of which was reported to highly express the adherens and TJ-proteins VE-cadherin, ZO-1, occludin, and claudin-5 [17].

Recently, our report on Tumor Treating Fields (TTFields), an FDA-approved treatment modality for glioblastoma [18,19,20] and mesothelioma [21], stated that there could be an increase in the permeability of the BBB in vitro and in vivo, an effect regulated by a Rho kinase-mediated claudin-5 phosphorylation pathway [22]. TTFields are electric fields of low intensity (1–3 V/cm) and intermediate frequency (100–500 kHz). They exert biophysical forces that disrupt cellular processes critical for cancer cell viability and tumor progression, ultimately leading to cell death [23,24,25].

In this study, we examined the effects of TTFields on the BBB in a 3D co-culture model consisting of primary human brain microvascular endothelial cells (HBMVEC) and immortalized human pericytes. Additionally, in this work, it was proven that 100 kHz TTFields can temporarily increase BBB permeability in an in vitro model of human origin.

## 2. Materials and Methods

### 2.1. Cell Culture and Maintenance

The HBMVEC were derived from a 26-year-old Caucasian female (iXCells Biotechnologies, San Diego, CA, USA) [26] and maintained in culture for up to eight passages, using endothelial cell medium and supplements (ScienCell Research Laboratories, Carlsbad, CA, USA). Meanwhile, immortalized pericytes (Celther, Lodz, Poland) were cultured in Dulbecco’s Modified Eagle’s Medium (DMEM) (Sigma-Aldrich, St. Louis, MO, USA) supplemented with 50 U/mL penicillin/streptomycin, 10% fetal calf serum (FCS), and 5 mL pericyte growth supplement (ScienCell Research Laboratories™) in a 37 °C incubator (Forma™ Steri-Cult™ 200, Thermo Fisher Scientific, Waltham, MA, USA) with 5% CO_2_ and 95% humidity confluent.

The cells were passaged in a 1:3 ratio weekly by dissociation with 0.25% Trypsin-EDTA (Gibco Thermo Fisher Scientific), and the medium was changed three times per week. Cells were controlled for mycoplasma contamination monthly using the Venor^®^ GeM Classic Mycoplasma Detection Kit for conventional PCR (Minerva BioLabs, Berlin, Germany).

Prior to experiments, HBMVEC were seeded at a density of 4 × 10^4^/cm^2^ onto transwell inserts with a pore diameter of 0.4 µm, pre-coated with 0.1% collagen IV (Sigma-Aldrich), and lodged in 24-well plates. Pericytes were seeded at a density of 2 × 10^4^/cm^2^ onto 20 mm diameter glass cover slips (A. Hartenstein, Würzburg, Germany) pre-coated with 0.5% gelatin, laid in 6-well plates (Greiner Bio-One, Kremsmünster, Austria). The cells were individually cultured on transwell inserts and cover slips for four days. Afterwards, the cells were set together by transferring the HBMVEC cultured in transwell inserts to the wells with pericytes, and they were then co-cultured for another four days. On day 9, experiments were started.

### 2.2. TTFields Application

The cells were set together in high ceramic dishes (Novocure^®^, Haifa, Israel) after individual culture in a 37 °C incubator Forma™ Steri-Cult™ 200 (Thermo Fisher Scientific), as described above. Next, the co-cultures were subjected to TTFields application (100–300 kHz frequency) using the inovitro™ TTFields Lab Bench System (Novocure^®^) for 24–96 h, as described previously [22]. Additionally, following treatment, cells were allowed to recover in the 37 °C incubator for 24–96 h.

### 2.3. Cell Counting

In counting cells before seeding and after treatment, the cells were washed twice with phosphate buffered saline (PBS) (Sigma-Aldrich) and dissociated with 0.25% Trypsin-EDTA (Gibco Thermo Fisher Scientific). Upon trypsinization, cells were vortexed and loaded onto the Scepter 2.0 Cell Counter device (Merck Life Science, Darmstadt, Germany) for counting.

### 2.4. Tight Junction and Associated Protein Expression Analysis

The expression of the TJ proteins claudin-5, ZO-1, occludin, and PECAM-1 was assessed via immunofluorescence staining and Western blotting as described previously [22,27].

#### 2.4.1. Immunofluorescence Staining

Subsequent to TTFields treatment, cells were washed three times with PBS and fixed in pre-chilled methanol for 20 min at −20 °C. Next, cells were washed again and blocked in 5% donkey serum (DS, Abcam, Cambridge, UK) diluted in PBS for 1 h at room temperature. The cells were afterwards incubated with the primary antibodies mouse anti-claudin-5 conjugated to Alexa Fluor 488 (1:500, Thermo Scientific, Cat. No. 352588), mouse anti-zonula occludens-1 conjugated to Alexa Fluor 488 (1:500, Thermo Scientific, Cat. No. 339188), and rabbit anti-PECAM-1 (1:500, Novus Biologicals, Centennial, CO, USA, Cat. No. NB100-2284) in 1% bovine serum albumin (BSA)/PBS with 5% DS overnight at 4 °C. On the following day, the cells probed with rabbit anti-PECAM-1 were probed with the secondary antibody anti-rabbit Alexa Fluor 555 (1:400 in 1% BSA/PBS with 5% DS, Invitrogen, Cat. No. A-21429) for 1 h at room temperature. In addition, the cells were then washed three times with PBS. Finally, the cover slips were mounted on glass microscope slides using Fluoroshield mounting medium with DAPI (Abcam), allowed to dry, and subsequently viewed under the microscope. Five representative fields of view per slide were photographed with the LEICA DMI 3000 B microscope, LEICA DFC 450 camera, and LAS V4.5 software (all Leica Microsystems, Wetzlar, Germany) with standardized settings at 40× magnification.

#### 2.4.2. Western Blot Analysis

The cells were washed twice with PBS and lysed with RIPA buffer (50 mM Tris pH 8.0, 150 mM NaCl, 0.1% SDS, 0.5% sodium deoxycholate, 1% NP40) containing protease inhibitor cOmplete ULTRA Tablets Mini (Roche, Basel Switzerland) and phenylmethylsulfonylfluoride (PMSF, Sigma-Aldrich). Additionally, after sonication (Sonopuls, Bandelin, Berlin, Germany) and determination of protein concentration (Qubit Protein Assay Kit, Life Technologies), samples were mixed with Laemmli buffer containing 5% β-mercaptoethanol (Sigma-Aldrich) and denatured at 95 °C for 10 min. 10 µL of PageRuler Plus Prestained Protein Ladder (Thermo Fisher Scientific) was loaded as a marker. The samples were run through a 10% SDS-PAGE minigel and blotted overnight using a Mini Trans-Blot Electrophoretic Transfer Cell (Bio-Rad, Hercules, CA, USA). Subsequently, the membrane was blocked in 5% non-fat dry milk (Carl Roth, Karlsruhe, Germany) and probed with the primary antibodies for claudin-5 (1:500, Invitrogen), PECAM-1 (1:500, Novus Biologicals), occludin (1:500, Invitrogen), and ZO-1 (1:500, Invitrogen), followed by a secondary anti-mouse or anti-rabbit antibody (1:3000, Roche Lumi Light Plus). Further, the horseradish peroxidase-conjugated anti-β-actin antibody (mouse) (1:2500, Sigma-Aldrich, Cat. No. A3854) served as an endogenous control. The detection was carried out using an enhanced chemiluminescence solution and viewed with ImagenFlourChem FC2 (Cell Biosciences, Preston, Australia) with the AlphaView Software (Version 1.3.0.7, Alpha Innotech Corp., San Leandro, CA, USA). The densitometric analysis was carried out using ImageJ (NIH) [28].

#### 2.4.3. Cellular Fractionation

To aid in visualizing alterations in claudin-5 localization, whole cell lysates were fractionated using the Sub-cellular Protein Fractionation Kit (Thermo Fisher) according to the manufacturer’s instructions. Additionally, cells were dissociated using 0.25% trypsin-EDTA (Gibco Thermo Fisher Scientific) and pooled in 2 mL microcentrifuge tubes (Eppendorf, Hamburg, Germany). Next, they were washed with PBS and centrifuged at 500× *g* for 3 min to obtain the cell pellet. The reagents were added, followed by incubation and centrifugation steps to acquire the cytoplasmic and membrane fractions.

### 2.5. Transendothelial Electric Resistance (TEER) Measurements

The cells were grown on 24-well PET transwell inserts (Corning, New York, NY, USA) with a pore diameter of 0.4 μm. Following the TTFields treatment, transendothelial electric resistance (TEER) was measured on top of a warm plate set to 37 °C with the volt-ohm meter device EVOM (World Precision Instruments, Sarasota, FL, USA). The blank filters served as internal controls.

### 2.6. Fluorescein Sodium Permeability Assay

After performing the TTFields experiments, the transwell inserts were transferred to a 24-well plate. The cells were washed with PBS, and 500 µL medium was pipetted into the wells. Next, 200 µL fluorescein sodium (Sigma Aldrich) dissolved in the same medium was added to the insert. A 100 μL medium was taken out from each well after 1 h for measurement of fluorescence intensity at a wavelength of 485 nm and 535 nm excitation and emission, respectively, using a Tecan GENios Microplate Reader (MTX Lab Systems, Vienna, VA, USA).

### 2.7. Statistical Analysis

The statistical analysis was performed with GraphPad Prism 8 software (GraphPad Software, San Diego, CA, USA) to determine significance using unpaired *t*-tests or one-way ANOVA. *p* < 0.05 was considered to be statistically significant.

## 3. Results

### 3.1. HBMVEC Co-Culture with Human Pericytes Increased Barrier Properties

**Figure 1:**
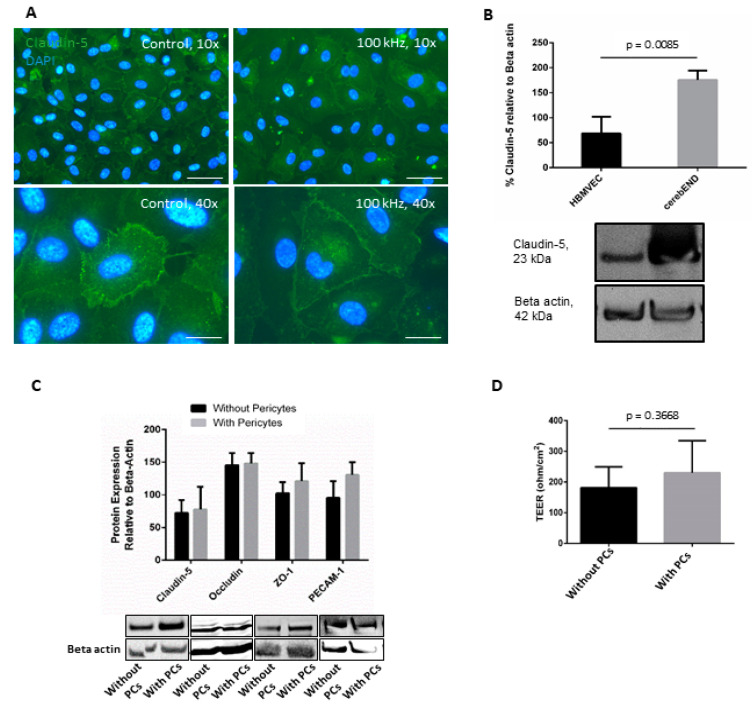
**The addition of pericytes to culture increased HBMVEC barrier properties.** (**A**) HBMVEC monoculture subjected to TTFields at 100 kHz for 72 h. Images shown are representative of at least three independent experiments. Scale bar = 200 µm (**B**) Western blot and densitometric analysis thereof, showing the difference of claudin-5 expression in untreated HBMVEC and cerebEND, n = 3. (**C**) Expression of tight junctional proteins in HBMVEC cultured alone or in combination with human pericytes (PCs) was demonstrated via Western blot and corresponding densitometric analyses, n = 3. The western blots shown are representative of at least three independent experiments. (**D**) Transendothelial electrical resistance (TEER) measurements of HBMVEC cells in monoculture or co-culture with pericytes, n = 6. Error bars represent the standard deviation.

Similarly, as in the case of cerebEND cells [22], we initially examined the effects of TTFields on HBMVEC as a monoculture. After treatment with 100 kHz TTFields for 72 h, a slight alteration in the distribution and localization of claudin-5 was observed via immunofluorescence staining (Figure 1A). However, compared to what we previously observed in cerebEND, which in itself showed a distinct staining of claudin-5 and a notable delocalization from the cell borders to the cytoplasm [22], distinct staining of claudin-5 in HBMVEC was inferior for both TTFields-treated and untreated conditions. Moreover, comparing HBMVEC and cerebEND using Western blots demonstrated a lower level of claudin-5 expression in the latter (Figure 1B).

Subsequently, the decision was taken to co-culture HBMVEC with immortalized human pericytes. Pericytes are components of the BBB and induce BBB characteristics such as barrier function and inflammatory responses [29]. As such, they are known to be involved in the buildup and expression of TJ proteins in endothelial cells [30,31].

The Western blot and corresponding densitometric analyses showed that the addition of pericytes increased ZO-1 and PECAM-1 expression, although no notable increase in the expression of claudin-5 or occludin was observed (Figure 1C). In addition, since barrier integrity is a primary characteristic of the intact BBB, we examined this in vitro by measuring TEER. Co-culture of HBMVEC with human pericytes demonstrated increased TEER (Figure 1D). Further, all succeeding experiments described in the following sections used HBMVEC in co-culture with human pericytes as a 3D model of the BBB.

### 3.2. TTFields at 100 kHz Frequency Altered Localization of Junctional Proteins Claudin-5 and ZO-1

**Figure 2:**
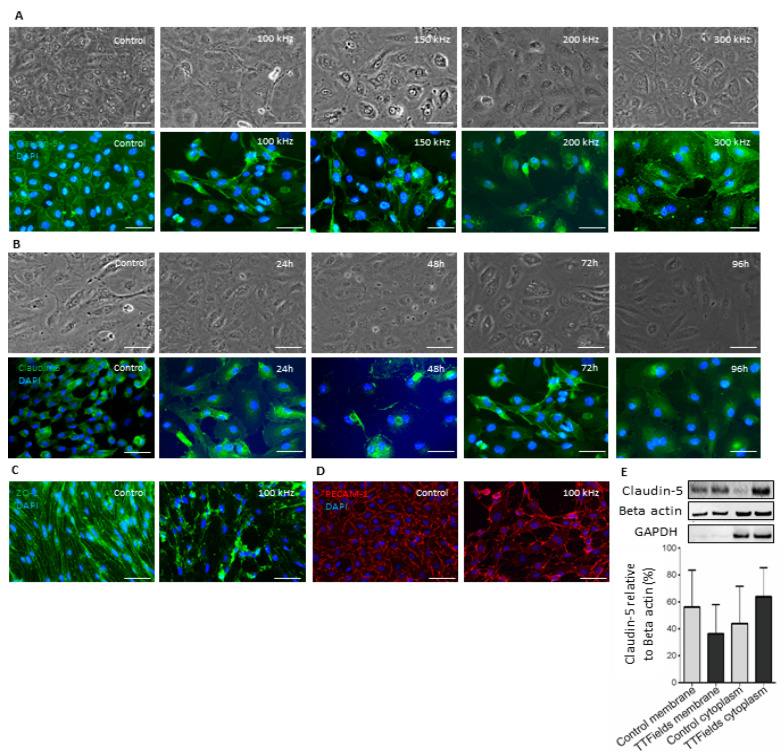
**TTFields altered the localization of tight junctions and associated proteins.** (**A**) Light microscope images of HBMVEC cells co-cultured with human pericytes (**upper panel**) and immunofluorescence staining of claudin-5 in the same cells (**lower panel**) treated with 100–300 kHz TTFields for 72 h. (**B**) Light microscope images of HBMVEC cells co-cultured with human pericytes (**upper panel**) and immunofluorescence staining of claudin-5 in the same cells (**lower panel**) treated with 100 kHz TTFields for 24–96 h. (**C**,**D**) Immunofluorescence staining of (**C**) ZO-1 and (**D**) PECAM-1 in HBMVEC subjected to TTFields at 100 kHz for 72 h. Images shown are representative of at least three independent experiments. Scale bar = 200 µm. (**E**) Western blot and densitometric analysis thereof of claudin-5 expression in fractionated HBMVEC, n = 3. Error bars represent the standard deviation.

The immunofluorescent staining of control HBMVEC 3D models revealed the localization of the TJ proteins claudin-5 (Figure 2A,B), ZO-1 (Figure 2C), and PECAM-1 (Figure 2D) along the borders of the cells. Additionally, upon subjecting the model to TTFields at 100–300 kHz for 24 to 96 h, the localization of the aforementioned proteins was altered. The most prominent effects were observed at 100 kHz (Figure 2A) for 72 h (Figure 2B), as demonstrated for claudin-5. Further, under these conditions, similar delocalization patterns were demonstrated also for ZO-1 (Figure 2C) and PECAM-1 (Figure 2D), though the effect for the latter was not as prominent as for the two other examined proteins. The Western blot confirmed the delocalization of claudin-5 in fractionated samples (Figure 2E). In the controls, claudin-5 was mostly present in the membrane fraction compared to the cytoplasmic fraction. However, in HBMVEC subjected to TTFields, the percentage of expressed claudin-5 in the membrane fraction decreased, whereas that of the cytoplasmic fraction increased.

### 3.3. Application of TTFields Transiently Diminished Barrier Integrity and Enhanced Permeability

**Figure 3:**
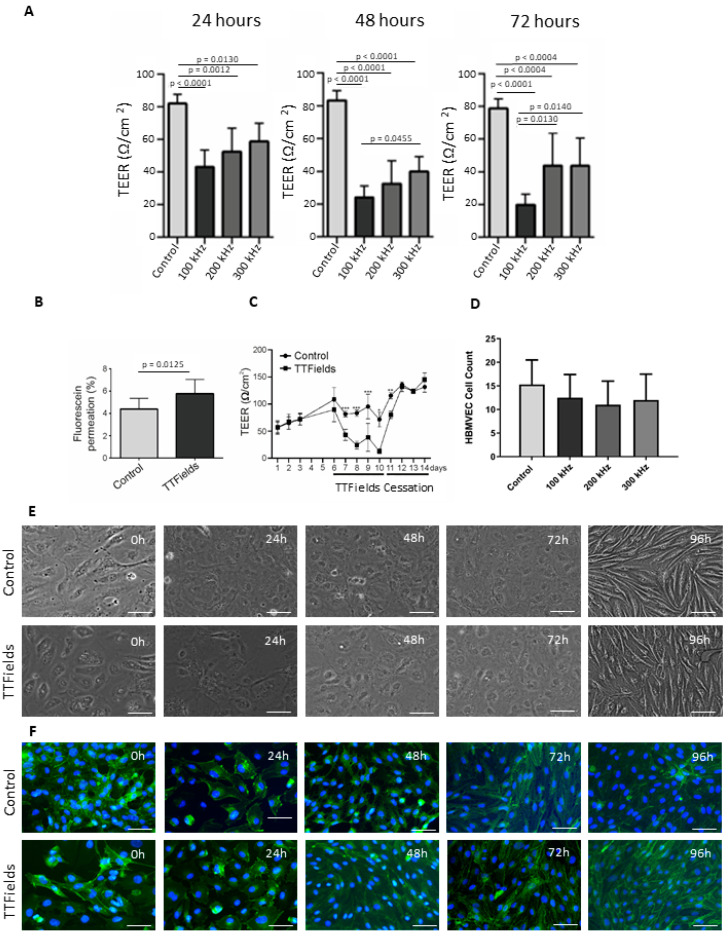
**TTFields exhibit transiently diminished barrier integrity and enhanced permeability.** (**A**) Transendothelial electrical resistance (TEER) measurements of HBMVEC cells treated with 100–300 kHz TTFields for 24–72 h, n = 6. (**B**) Permeation of fluorescein sodium in HBMVEC with or without TTFields treatment. (**C**) TEER measurement of HBMVEC before TTFields, post-TTFields, and after a period of recovery once TTFields administration was ceased. Asterisks indicate significant differences. *p*-values * < 0.05, ** < 0.01, *** < 0.001. (**D**) HBMVEC cell count after treatment with 100–300 kHz TTFields for 72 h. (**E**) Bright Field Microscopy of HBMVEC cells post-TTFields and (**F**) Immunofluorescence Staining thereof to visualize claudin-5. Cells were treated with TTFields at 100 kHz for 72 h before allowing them a recovery period of 24–96 h. Images shown are representative of at least three independent experiments. Scale bar = 200 µm.

In the application of TTFields to our 3D model, the most significant reduction in TEER was observed when applying TTFields at 100 kHz. At all examined time points (24–72 h), TEER values at all tested frequencies (100–300 kHz) were significantly reduced compared to the control. However, the most significant difference was demonstrated at 72 h where 100 kHz showed reduced TEER that was significantly different from both 200 and 300 kHz (Figure 3A). Additionally, at 100 kHz TTFields frequency, TEER measurements of HBMVEC showed an increased effect over time, with TEER values at 48 and 72 h significantly reduced compared to those at 24 h (Figure 3A). The TTFields-induced decline in TEER was further confirmed by a significantly increased permeation of fluorescein sodium in HBMVEC, to which 100 kHz TTFields were administered for 72 h (Figure 3B).

Additionally, after subjecting the cells to TTFields, they were made to recover at 37 °C for a period of 24–96 h. The drop in TEER observed after TTFields application gradually reverted to values similar to those of the control after a period of recovery once TTFields were ceased (Figure 3C). However, despite these changes, there was no significant alteration in the number of cells (Figure 3D). The disrupted cells started to regain their normal morphology already after 24 h, but complete recovery, as demonstrated by TEER values and the visual appearance via immunofluorescence staining of claudin-5, was reached after 48 h (Figure 3E,F).

## 4. Discussion

Recently, a study was conducted that described the effects of TTFields on murine cerebellar microvascular endothelial cells in vitro and in vivo in healthy rats. The findings demonstrated that 100 kHz TTFields applied for 72 h increased BBB permeability by delocalizing TJ proteins such as claudin-5 through a Rho-kinase-dependent pathway. In addition, TTFields alter the microtubular organization, activating GEF-H1, which thereby leads to increased RhoA levels. The ROCK is then activated, resulting in claudin-5 phosphorylation, which causes the delocalization of claudin-5 from the cell borders to the cytoplasm [22]. Further, ZO-1 and occludin localization were also altered. It was therefore our goal to validate the effects in an in vitro human cell-based system.

Studies that aim to reach clinical translation require rigorous pre-clinical assessment. Pre-clinical studies call for investigations using in vitro and in vivo animal models. Although in vivo models provide the advantage of assessing effects at the organismal level, in vitro models allow the construction of a biological system that enables investigations limited by in vivo systems [32]. The in vitro model of choice is crucial to the success of its translation to the clinic. However, not only the type and construction of a model need significant consideration, but also the selection of the cell source. The consideration of species-specific effects and their ability to faithfully resemble effects in humans is critical, as translational experiments have failed due to differences between species [33,34]. Thus, in general, the responses of human-derived cells would more closely simulate in vivo conditions in patients compared to those derived from other species. Therefore, although we were already able to show promising preliminary results using murine models, we opted to verify our findings in a human cell-based transwell co-culture model made up of primary human microvascular brain endothelial cells [26] and immortalized pericytes. A non-contact co-culture system was used for this validation to facilitate better handling with regards to staining of endothelial tight junctions.

In the model presented, we preferred to use only one other cell entity, i.e., pericytes, and not pericytes together with astrocytes or even also neurons, since we already were able to visualize not only better staining of tight junctional proteins upon co-culture of endothelial cells with pericytes but also observed higher TEER values. The initial aim was to validate the effects of TTFields that were observed in murine in vitro and in vivo models in a human-based system. However, because our murine in vitro model was only comprised of a monolayer, we first and foremost conducted validation experiments using HBMVEC monolayers. Further, in using only monoculture, staining of tight junctions and the low TEER values presented a challenge. We only achieved better morphological visualization and a higher TEER when we added pericytes to the culture. Further, since this was the case and the addition of pericytes sufficed for our aims, we have not included other cellular entities of the neurovascular unit such as astrocytes and neurons anymore. Nonetheless, the use of a triple or even quadruple co-culture of HBMVEC, pericytes and/or astrocytes/neurons, is considered for future experiments.

The most widely used in vitro model of the BBB is based on co-culture of the BBB component cells seeded on transwell inserts lodged onto well plates. This type of model replicates in vivo conditions more precisely compared to monocultures without requiring stringent handling methods [33,35]. The co-culture of endothelial cells with pericytes proved to be a stable and useful BBB model [36,37,38]. Reports on the use of human brain microvascular endothelial cells such as hCMEC/D3 in monoculture as a BBB model abound in the literature [38,39,40]. However, it is known that monolayers of hCMEC/D3 cells form only moderately restrictive barriers, most likely because the major tight junction protein, claudin-5, is markedly downregulated [41]. Therefore, we opted to use another cell line, HBMVEC, in combination with human pericytes, since the interaction of pericytes with endothelial cells fulfills a critical function in the regulation of numerous signaling pathways [42,43]. The multiple rat, porcine, and human cell-based models demonstrated that TJ proteins and transporter proteins such as transferrin and P-glycoprotein are increased in endothelial cells co-cultured with pericytes and/or astrocytes [14,44]. Similar observations were made in the in vitro model as the TEER of HBMVEC increased and the staining of claudin-5 became more distinct with pericyte co-cultivation. Nonetheless, it should be noted that the HBMVEC we used in this study is derived from a Caucasian female [26]. It has been reported that adhesion and proliferation yield in the culture of human brain microvascular endothelial cells do not only depend on the brain region from which the cell was derived but are also gender-specific [45]. It was recently reported that TTFields have varying effects in different ex vivo samples derived from male and female glioblastoma patients [46]. In addition, it is worth mentioning that appropriate immunodetectable junctional molecules might be used as sensitivity markers for normal or abnormal BBB function. Further, it was reported that in human brain microvessels, the interendothelial junctional complexes contain molecular components specific for both tight and adherens junctions [47].

The previous findings in murine in vivo and in vitro models were replicated and thus verified in this current 3D human cell-based model. In addition, as shown thus far, all frequencies (100–300 kHz) and durations of TTFields application demonstrated effects, but the most optimal and significant was at 100 kHz for 72 h. The application of 100 kHz TTFields for 72 h increased the permeability of the BBB model, as shown by delocalization of TJ proteins, increased permeability, and decreased TEER, as was also previously shown in the murine model. The only difference we observed was the shorter recovery period of 48 h needed by HBMVEC post-TTFields, compared to 96 h in cerebEND. This variation may be due to species-specific characteristics, brain region-specificity, or gender-specificity [45].

Finally, after the cessation of TTFields, the cells were able to gradually recover as the microtubular organization was regained. The TTFields depolymerize the microtubules, allowing for structural reorganization that leads to activation of GEF-H1, ultimately leading to claudin-5 phosphorylation and subsequent delocalization. Further, while the cells recover, the effects of TTFields are reversed [22]. The increased permeability and decreased TEER observed upon TTFields administration show altered BBB integrity, which is important for enabling chemotherapeutics and other substances to reach the target site. The effects are non-cytotoxic, as shown by stable cell counts at all frequencies tested, and the ability of the BBB to recover from the effects proves significant for a transient BBB opening.

## 5. Conclusions

Overall, using this model, we could validate that TTFields transiently alter cellular junction localization in human brain microvascular endothelial cells, leading to enhanced BBB permeability. Moreover, with this new data, we are one step closer to implementing a novel method to open the BBB, which could aid in the treatment of numerous central nervous system disorders.

## 6. Patents

Using alternating electric fields to increase the permeability of the blood-brain barrier has been patented with Novocure as the patent applicant and C.H., M.L., A.F.K., and M.B. as inventors (US Appl. Nos. 63/015,099 and 63/071,748).

## Data Availability

The authors confirm that the data supporting the findings of this study are available within the article. Raw data are available from the corresponding author upon reasonable request.
